# Investigation of metal mobility in gold and silver mine tailings by single-step and sequential extractions

**DOI:** 10.1007/s10661-022-10054-3

**Published:** 2022-05-12

**Authors:** Paramee Kumkrong, Eben Dy, Daniel D. Tyo, Cindy Jiang, Indu Gedara Pihilligawa, David Kingston, Patrick H. J. Mercier

**Affiliations:** 1 National Research Council Canada, 1200 Montreal Road, Ottawa, ON K1A 0R6 Canada; 2 Corem, QC Canada

**Keywords:** Dissolution, Extraction, Mobilization, Precipitation, Oxidation, Reduction, Sequential extraction

## Abstract

Metal leachate from mine tailings has the potential to release toxic metals into the surrounding environment. A single-step extraction procedure mimicking rainwater and a three-step BCR sequential extraction procedure (acid, reducing and oxidizing conditions) were applied to gold (GMT) and silver (SMT) mine tailings. Major (Al, Ca, Fe, Mg, and Mn) and trace metals were monitored to better understand the mobility and geochemistry of these metals when exposed to various environmental leaching conditions. Rainwater extraction released only small quantities of metals, while the three-step BCR extraction was more effective in mobilizing metals from the tailings. Under the acidic conditions of BCR step 1, Ca, Mg, Cd, Cu, and Mn were released in high concentrations. The dissolution of Fe, Ca, and Mg were dominant along with Pb in step 2 (reducing conditions). In step 3 (oxidizing conditions), Fe was the most dominant species together with Co, Cu, Ni, and Se. A high fraction of Al, Be, Cr, Li, Mo, Sb, Tl, and V remained in the residue. From SMT, larger quantities of As, Ca, Cd, and Zn were released compared to GMT. The BCR extraction could be applied to tailings to predict the potential release of toxic metals from mine wastes; however, excessive amounts of Ca and Fe in the tailings could cause carry-overs and incomplete extraction and carry-overs, resulting in a misinterpretation of results.

**Supplementary information:**

The online version contains supplementary material available at 10.1007/s10661-022-10054-3.

## Introduction

Every year, the mining industry produces over one hundred million metric tonnes of solid waste in Canada (Statistics Canada, 2012). A large portion of this solid waste (waste rocks and tailings) has the potential to release toxic elements into the surrounding environment and impact water chemistry and biology. Tailings are defined as wastes after ore extraction and consist of fine particles with large surface area and high adsorption capacity (Kermani et al., 2015). Tailings are transported by wind, precipitation, and runoff water and deposited in both terrestrial and aquatic environments. As a result, areas near mining facilities could show elevated concentrations of toxic metals in topsoil (García-Giménez & Jiménez-Ballesta, 2017; Schuh et al., 2019), water (Kumar et al., 2021; Palmer et al., 2021; Shaw et al., 2011), and river sediments (Clark et al., 2021; Sánchez-Donoso et al., 2021; Schuh et al., 2018), raising concern about contaminating habitat and food webs (Nawab et al., 2015; Wang et al., 2021; Xiao et al., 2008).

Solid mine wastes are heterogeneous materials often containing various sulfide minerals. Sulfide minerals such as pyrite (FeS_2_), pyrrhotite (Fe_(1-x)_S), galena (PbS), sphalerite ((Zn,Fe)S), and chalcopyrite (CuFeS_2_) are reactive species (Jamieson, 2011; Martín-Crespo et al., 2019). Previous studies on metal leachates reported high amounts of toxic elements mobilized from sulfide mineral tailings (Fan et al., 2016; Schaider et al., 2007). Long-term environmental exposure of tailings via natural weathering contributes to the oxidization of sulfide minerals and generates acid rock drainage (ARD) which later dissolves the mineral phases resulting in metal release (Dold, 2014; Gunesegeran et al., 2021; Jamieson et al., 2015). Released metals entering the biota poses the risk of accumulation and biomagnification. Metal leachates can be investigated along with the total metal concentrations in tailings as a useful tool to assess and potentially mitigate the risks to the environment (Kumpiene et al., 2017).

The synthetic precipitation leaching procedure (SPLP), known as the EPA 1312, is a single-step extraction method by the Environmental Protection Agency (EPA, 1994) and used for solid waste characterization. The precipitation simulates slightly acidic (pH 4.2) rainwater, which is derived from the reaction of moisture, sulfur dioxide, and nitric oxide in the atmosphere.

Over the years, various sequential extraction procedures have been explored for sediments targeting specific mineral phases and elements (Barber, 1974; Gleyzes et al., 2002; Piatak et al., 2007; Song et al., 2015). The BCR sequential extraction procedure has been established by the European Commission Community Bureau of Reference (BCR) (later renamed as Standards Measurements and Testing Program (SM&T)) and is in worldwide use. The advantage of the BCR procedure over other sequential extraction approaches is the availability of certified reference material (CRM) BCR 701 lake sediment (Pueyo et al., 2001). The CRM is typically used for quality control and method verification for sediment extraction. The application of the BCR procedure has been growing and has expanded to other solid samples of environmental interest (Kumkrong et al., 2021a, b; Rauret et al., 2001).

The BCR procedure is a three-step extraction using various extractants to access specific mineral compartments:

- Step 1, exchangeable, carbonate, and halite minerals
- Step 2, Fe oxy-hydroxide minerals
- Step 3, organic matter  

The residual fraction after extraction of BCR represents elements that are intact in primary sediments (minerals formed during the original crystallization of the host igneous primary rock) and considered as non-mobile elements and present only a marginal risk to the surrounding environment. Furthermore, the residue fraction is used in recovery and mass balance calculations to evaluate the performance of the BCR extraction (the combined concentrations of three-step extraction and residue vs total metal concentration) (Kumkrong et al., 2021a, b; Sutherland, 2010).

In this study, a single-step extraction based on synthetic precipitation (rainwater) modified from EPA 1312 and the BCR sequential extraction was applied to gold and silver mine tailings from Canada. Sixteen trace elements regulated in waters under Canadian water quality guidelines (including As, Be, Cd, Co, Cr, Cu, Li, Mo, Ni, Pb, Sb, Se, Tl, U, V, and Zn) (*Canadian Council of Ministers of the Environment | Le Conseil Canadien Des Ministres de l’environment*, 2021), along with major metals (Al, Ca, Fe, Mg, and Mn), were determined to assist the interpretation of the dissolution of mineral phases. The risk assessment code (RAC) was modified to investigate the tailings (Sarkar et al., 2014).

## Materials and methods

### Samples

Tailing samples were collected from gold and silver mine tailings in Canada. The samples from the gold mine tailing were identified as GMT, and the sample from the silver mine tailing was identified as SMT. The samples were stored in water to prevent oxidation during storage, sub-sampled, and air-dried in a fume hood. After air-drying, the samples were ground and used for the leaching studies.

### Laboratory ware

Laboratory ware for the extraction consisted of a 50-mL polypropylene conical tube, pre-washed by soaking in 10% v/v nitric acid (HNO_3_) overnight, and rinsed with deionized water (DIW).

### Chemicals and standards

All chemicals were analytical grade or better and acquired from Millipore Sigma, Canada. DIW was produced in-house using Millipore Synergy UV-R.

The individual 1000 mg L^−1^ standard solutions of Al, As, Ca, Co, Cr, Cu, Fe, Li, Mg, Mn, Ni, Pb, Se, V, and Zn were ICP grade and acquired from SCP Science; Be, Cd, Mo, Sb, and Tl were from ISO Spec; and U was from High-Purity Standard. All standard solutions were traceable to a SI via NIST as claimed by the providers. The working standard solutions were prepared in three sets: 1 to 10 mg L^−1^ of major metals (Al, Ca, Fe, Mg); from 0.1 to 3 mg L^−1^ for As, Co, Cr, Cu, Mn, Pb, Sb, V, and Zn; and from 0.001 to 0.1 mg L^−1^ for Be, Cd, Li, Mo, Se, Sb, U, and Tl. All standard working solutions were prepared in 2% v/v nitric acid (HNO_3_). PACS-3 marine sediment CRM from NRC Canada (Willie et al., 2013) was used for quality control for elemental analysis and BCR 701 CRM for the sequential extraction (certified for Cd, Cr, Cu, Ni, Pb, and Zn).

### Instrumentation

The determination of elemental concentration was conducted using an inductively coupled plasma optical emission spectrophotometer (ICP-OES), Varian 720, Agilent Technologies Inc., Australia. When the concentration was below the detection range for ICP-OES, an inductively coupled plasma mass spectrometer (ICP-MS), Agilent 7900 with collision reaction cell and MassHunter software, Agilent Technologies Inc., USA, was used. The concentration of C, H, N, and S was analyzed using an elemental analyzer, Elementar Vario EL CUBE, Elementar Analysensysteme GmbH, Germany.

Scanning electron microscopy and energy dispersive spectroscopy (SEM–EDS), the Hitachi SU5000 analytical SEM microscope equipped with an Oxford Instruments X-MaxN 80 mm EDX spectrometer and backscattered electron (BSE) imaging, was used. EDX analysis was performed at an accelerating voltage of 20 kV and a live time of 60 s per analysis.

### Total digestion procedure for tailings and residues

Three acids (69% w/w HNO_3_, 37% w/w HCl, and 48% w/w HF) were used for microwave digestion (Anton Paar Multiwave PRO with eight digestion tubes, Anton Paar GmbH, Austria). The procedure is detailed in Supplementary information.

### Performance of total metal analysis using PACS-3 CRM

Recoveries were calculated by comparing measured and certified values and associated uncertainties in PACS-3 CRM (Willie et al., 2013). The average recovery for the 17 studied elements was 96% ± 10%, and the lowest observed recovery was 76% for Sb. The measured value and expanded uncertainty (coverage factor (*k*) = 2) of elements were within the certified range for all elements with the exception of Cu. The recovery of total concentrations after digestion is illustrated as a bar graph in Supplementary information Figure S1 and Table S2. There are no significant differences between the measured and certified values using the *t* test paired two-sample for means.

### Single-step extraction

One g of sample was used, and the solid-to-liquid ratio was 1 g to 20 mL. The tailing samples were placed in the extraction solution (pH 4.20 ± 0.05) and shaken for 18 h using a mechanical end-over-end shaker (Genie SI-1100 Roto-Shake Rotator, Scientific Industries Inc., USA) at a speed of 30 ± 10 rpm at 22 ± 5 °C. After extraction, the solution was separated using a centrifuge (Thermo Scientific Sorvall Legend XFR, Thermo Fisher Scientific, Germany) at a speed of 3000 rpm for 20 min. The supernatant was collected and acidified to pH 2 using 69% w/w HNO_3_ (EPA, 1994).

### BCR extraction

The BCR extraction procedure is described by Rauret et.al. (Rauret et al., 2001). The extraction reagent is mentioned in Supplementary information.

In step 1, the solid-to-liquid ratio was 1 g to 40 mL of 0.11 mol L^−1^ acetic acid. The extraction was performed in the same system as in the single-step extraction with 16 h extraction time. After the extraction, the supernatant was separated. The residue was washed with 20 mL of DIW, and the rinsed solution was discarded.

In step 2, 40 mL of 0.5 mol L^−1^ hydroxylamine hydrochloride in diluted HNO_3_, pH 1.4 ± 0.05 was added into the residue of step 1, and the extraction was processed in the same manner as in step 1.

In step 3, 10 mL of 30% w/w hydrogen peroxide (H_2_O_2_) was added to the residue of step 2. The tube was left at room temperature for pre-reaction for 1 to 2 h and later placed in a hot block at 85 ± 5 °C (Environmental Express Model SC100, Environmental Express Inc., USA). The heating was continued until the volume was reduced to 1 mL. The oxidation at high temperature was repeated in another cycle before adding 50 mL of 1 mol L^−1^ ammonium acetate at pH 2.0 into the tube and extracting for 16 h. The supernatant was separated for analysis. The residue was washed, dried (60 °C), and used for residue digestion.

The extracts were preserved at pH 2 by adding a few drops of 69% v/v HNO_3_ and kept in the refrigerator until analysis. Dilution, if required, was carried out using 2% v/v nitric acid. Reagent blank from each extraction step was used for blank subtraction.

### Performance of the BCR extraction on BCR 701

The overall average recovery from step 1 to step 3 using BCR 701 CRM was 95% ± 11%. There is no significant difference between the measured values and certified values, except for the low recovery of Cr in step 2. The performance of the BCR extraction on the BCR 701 CRM is presented in Supplementary information Figure S2.

### Performance of the BCR extraction on tailings

The combined concentrations of three-step extraction and residue were compared against the total concentration of metals in the tailings and reported as percent recoveries (Supplementary information Tables S2 and S3). The recovery (%) of the extraction ranged from 70% (Sb) to 126% (Se) with an average of 96% ± 15% for GMT and from 74% (Li) to 99% (Tl) with an average of 88% ± 8% for SMT. A low recovery for some elements was likely the result of losses during the rinse and the violent reaction of sulfide minerals in step 3.

### Data analysis

An approach of using a percent-released fraction (Eq. 1) to access the degree of dissolution was modified from the risk assessment code (Chen et al., 2018; Sarkar et al., 2014). Metals with more than 50% extracted from the mineral phase into the solution phase are considered an extremely high release (mobile); 31% to 50% is high release; 11% to 30% is moderate release; 1% to 10% is low release; and below 1% is considered no release and the least mobile elements:

1 $$\begin{aligned}Fraction \left(\mathrm{\%}\right)=\frac{{\left[X\right]}_{i} x 100}{{\sum }_{i=1}^{3}{\left[X\right]}_{i}+{\left[X\right]}_{residue}}\end{aligned}$$

where [*X*]_i_ is the concentration (mg kg^−1^) of an element from step *i* (*i* is from 1 to 3).

## Results

### Mineralogy of tailings

SEM–EDS reveals that the studied tailing samples consist of fine particles ranging from 1 to 50 µm. GMT contained mainly two ranges of particles (bimodal distribution) from 25 to 50 µm and 5 to 25 µm. SMT particle sizes were smaller and ranged from 5 to 25 µm.

Mineral composition in both samples was similar and consisted of aluminosilicates of quartz (SiO_2_), albite (NaAlSi_3_O_8_), chlorite [(Mg,Fe)_10_Al_2_(Al_2_Si_6_O_20_)(OH)_16_], plagioclase feldspar [(Ca,Na)AlSi_2_O_8_], alkali feldspar [(K,Na)(AlSi_2_O_8_)], muscovite [K_2_Al_4_(Si_6_Al_2_O_20_)(OH,F)_4_], and biotite [K(Mg,Fe)_3_(AlSi_3_O_10_)(OH)_2_]. The majority of aluminosilicates in GMT were free of any fine-grained mineral coating, attached grains or surface alteration. In SMT, aluminosilicates were frequently coated with finer-grained aluminosilicates.

In GMT, pyrite (FeS_2_), rare-earth element (REE) phosphates, and rutile were observed as minor minerals at a fraction of 5 µm to 10 µm particle size attachments or inclusions on the larger aluminosilicates. In SMT, some arsenopyrite (AsFeS_2_), silver-bearing tetrahedrite [(Cu,Ag)_12_As_4_S_13_], galena (PbS), and sphalerite [(Zn,Fe)S] were also present. The BSE images of the two tailings and residue after step 3 extraction are in Supplementary information Figures S3 and S4.

As for the residue, the mineral composition of both tailings remained the same after single-step extraction. Pyrite in GMT was attached to the surface of aluminosilicates, and REE phosphates were attached to feldspars. In SMT, pyrite, pyrrhotite [Fe_(1-x)_S_x_], and iron oxides (either hematite (Fe_2_O_3_) or goethite (FeOOH)) were observed as incursions or attachments to the aluminosilicates.

After three-step extraction, the residual particle sizes of GMT and SMT were dominated by 10 µm to 25 µm, and fine-grained particles were no longer observed indicating complete dissolution. The grain surfaces from GMT were clean with very few fine-grained minerals attached. With SMT, a minor fraction of aluminosilicates was covered with finer-grained aluminosilicates, most likely sheet silicates such as chlorite or muscovite. Surprisingly, that pyrite was still observed together with very fine-grained REE phosphates after oxidative reaction.

### Elemental analysis

**Fig. 1:**
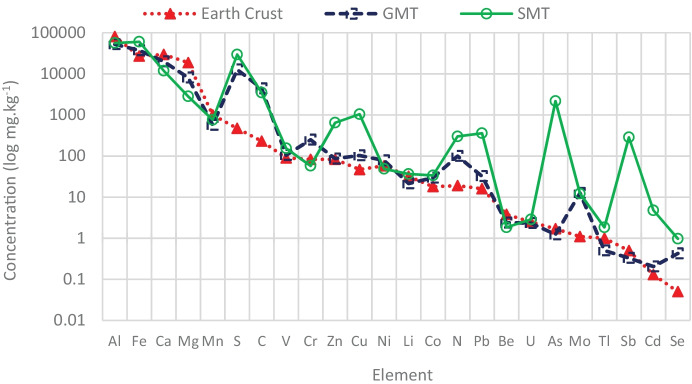
Concentrations of elements (log mg kg^−1^) in GMT and SMT tailings vs. earth crust (Yaroshevsky, 2006)

The total concentration of elements in tailings was determined and compared to the typical concentration in the earth crust (Yaroshevsky, 2006). In Fig. 1, the concentration of elements is in a log scale (mg kg^−1^) and arranged from high to low. It is noted that both tailings contained high amounts of S, C, N, Cu, Fe, Mo, and Se compared to the abundance of elements in the crust. The concentrations of elements in the tailings are presented in Supplementary information Tables S2 and S3. SMT contained two orders of magnitude higher levels of As, Cd, Cu, Pb, Sb, and Zn than GMT. GMT showed two to four times higher concentration of Cr, Mg, and Ni than SMT.

### Single-step extraction: precipitation

The mass fraction of metals from the single-step synthetic precipitation extraction is presented in Supplementary information Tables S2 (GMT) and S3 (SMT). Synthetic precipitation or rainwater (hereafter called precipitation) at pH 4.20 showed no effect on the pH of tailings, and only a slight change of pH was observed (GMT pH changed from 7.05 to 8.10 and SMT pH from 7.54 to 7.40), indicating a high buffering capacity of the tailings. Mild alkalinity is consistent with a high amount of Ca (2,311 mg kg^−1^ for GMT and 1,786 mg kg^−1^ for SMT) dissolved from the tailings, and possibly from exchangeable Ca(HCO_3_)^2−^ that loosely bound to the surface tailings, or from Ca(HCO_3_)_2_ that commonly forms when an excessive amount of Ca is exposed to air and moisture.

In the single-step extraction, small amounts of Mg (73 mg kg^−1^ to 109 mg kg^−1^) and trace amounts of Mn and Zn (1 mg kg^−1^ to 3 mg kg^−1^) were observed, suggesting that they were from either highly soluble salts of bicarbonate or exchangeable ions. Mobilization of other metals was marginal in these conditions (less than 2% of total concentrations). As such, the risk of toxic metals release to the environment as a result of precipitation (rain) is deemed relatively low, with the exception of Ca and Mg. Ca and Mg could possibly affect water chemistry by changing the pH and increasing water hardness. However, the release of 15% of total Mo content could be of concern for SMT.

### BCR sequential extraction

**Fig. 2:**
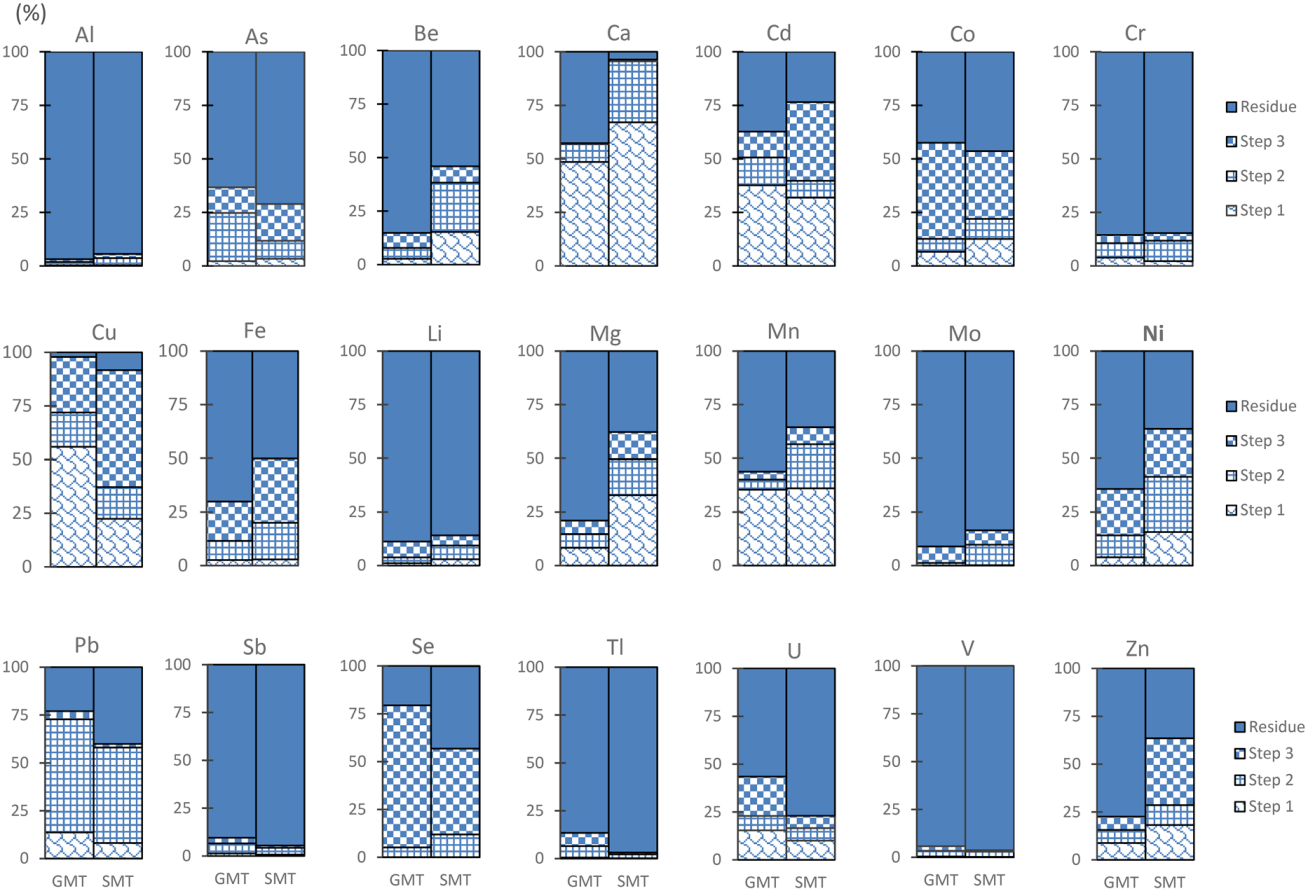
Distribution (%) of elements in gold (GMT) and silver (SMT) mine tailings by BCR sequential extraction steps and residue

All data on BCR extraction of GMT and SMT is presented in Supplementary information Tables S2 and S3. The released fractions (%) of metals from both tailings are presented as a stacked bar graph in Fig. 2.

#### Step 1

Using 0.11 M acetic acid, pH 2.96, the pH of the final extract of both tailings was shifted from neutral to pH 3.72 (SMT) and 3.95 (GMT), suggesting the tailing lost buffering capacity causing more dissolution of carbonate minerals. Ca was the most dissolved metal from both tailings (10,216 mg kg^−1^ in GMT and 7,102 mg kg^−1^ in SMT), approximately four times higher than the EPA precipitation extraction. Mg and Mn dissolved along with Ca. The released fractions of Ca (67%) and Mg (33%) in SMT were much higher than in GMT, implying the majority of Ca and Mg in SMT exist as soluble, carbonate, and sulfate minerals (Farmaki et al., 2018; Mester et al., 1998). High concentration of Fe (1,283 ± 440 mg kg^−1^) was released along with Ca, a phenomenon not observed in the precipitation extraction.

**Fig. 3:**
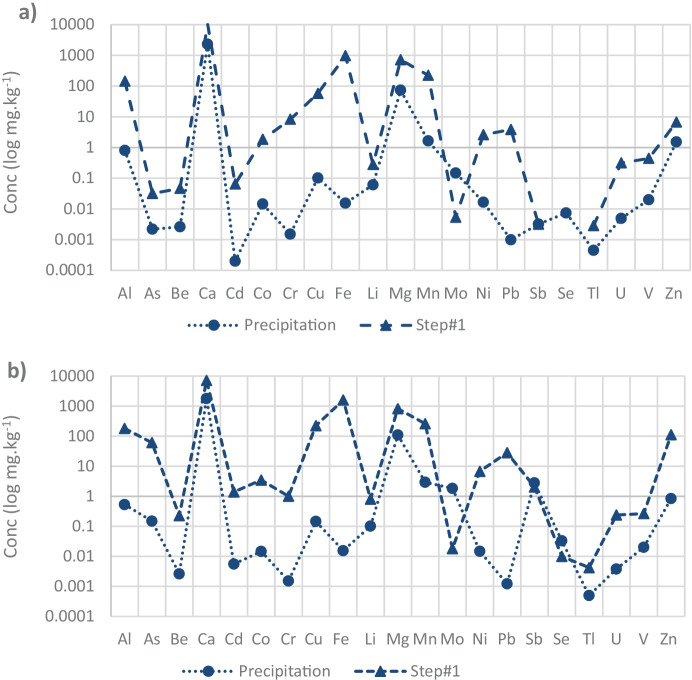
Concentrations of elements (log mg kg^−1^) by precipitation and BCR step#1 in a gold (GMT) and b silver (SMT) mine tailings

Other minor and trace metals that dissolved in large proportions (over 30%) from both tailings included Cu and Cd. Cu in SMT showed four times higher concentration (223 mg kg^−1^) than in GMT. Zn and As in SMT were dissolved in high concentrations (60.5 mg kg^−1^ and 111 mg kg^−1^, respectively). The dissolution by BCR step 1 extraction is at least 100 times higher than the precipitation extraction, with the exception of Mo, Sb, and Se (Fig. 3).

#### Step 2

In step 2, the pH of the extractant remained acidic (pH 1.57 to 1.88). As expected, the release of a high amount of Fe (3,300 mg kg^−1^ in GMT and 9,501 mg kg^−1^ in SMT) was observed. The reducing agent in acidic conditions dissolved the amorphous Fe oxy-hydroxide and Mn oxide minerals (Vodyanitskii, 2010). Interestingly, Al (a major metal present as aluminosilicates in the tailings as observed by SEM/EDX) was also observed in this fraction at high concentrations (856 mg kg^−1^ in GMT and 1,840 mg kg^−1^ in SMT), suggesting the dissolution of Al, possibly from Fe-Al-oxy-hydroxide minerals. This finding corroborates the observations of Piatak et al. (Piatak et al., 2007), who studied the dissolution of amorphous and crystalline Fe-Al hydroxide and Mn oxides in tailings from Vermont and Maine, using similar reagents. A surprisingly low concentration of Mn was observed in this study, implying Mn oxides are less abundant in Fe clay minerals of the two tailings (Piatak et al., 2007; Trifi et al., 2018). High amounts of Ca and Mg were still detected in step 2, possibly carried over from the step 1 of the sequential extraction. For better interpretation, the extraction by BCR step 1 could be conducted twice to ensure complete removal of carbonate minerals from the samples.

Apparently, Pb was dissolved with Fe and became a dominant fraction (greater than 50%), followed by Cu and Ni. For other metals, less than 0.5 mg kg^−1^ of Be, Cd, Mo, Tl, Se, and U were observed in both tailings. Metals of concern from SMT that dissolved with Fe-Al-oxy-hydroxide minerals at high concentrations were Pb (172 mg kg^−1^) > As > Cu >  > Zn >  > Sb >  > Cr (4.31 mg kg^−1^), whereas, these elements were significantly lower in GMT, except for Cr (14.5 mg kg^−1^).

#### Step 3

Extraction step 3 uses an oxidizing agent at elevated temperature to extract metals bound to organic matter. However, for tailing samples, the concentration of carbon was two to eight times less than the concentration of sulfur, (see Supplementary information Tables S2 and S3); therefore, the sulfide minerals were more dominant than carbon organic matter. A twice the concentration of Fe (6,850 mg kg^−1^ GMT and 16,637 mg kg^−1^ SMT) was observed compared to step 2 (reducing condition), suggesting Fe in both tailings is present as sulfide minerals. High concentration of Fe found in this step is consistent with the SEM/EDX observations, revealing the presence of Fe-sulfide minerals (pyrite-GMT, pyrite, galena, arsenopyrite, and sphalerite in SMT).

After step 3, we suspect high concentrations of both S and Fe still remained in the residue, potentially indicating that the reaction conditions of step 3 might be insufficient to extract the excessive amounts of sulfide minerals present in these samples (Warwick et al., 2006). Ca was observed below 100 mg kg^−1^ in step 3 implying Ca secondary minerals were mostly removed from the tailing after BCR steps 1 and 2. The observation of a small amount of Ca in this step is similar to heterogeneous marine sediments (Kumkrong et al., 2021; Kumkrong et al., 2021). Al and Mg were still observed at high concentrations at a similar level to step 2. The release of Al under oxidizing conditions was also observed in various types of sediments (Kryc et al., 2003; Kumkrong et al., 2021; Kumkrong et al., 2021; Piatak et al., 2007; Sutherland & Tack, 2002).

In Fig. 2, two tailings shared similar characteristics of high release fraction of Se (45% to 74%) > Co, Cu > Ni (22%), suggesting these elements were associated with Fe-sulfide minerals. SMT released higher amounts of As, Zn, and Cd compared to GMT.

#### Residue

The concentration of elements in the residue indicates a strong bond between metals and primary minerals. The SEM/EDX analysis revealed that the majority of tailings consisted of aluminosilicate minerals, and over 90% of Al was observed in the residue along with Sb and V. Additionally, high fraction (81% to 90%) of Cr, Li, Mo, and Tl remained in the residue of both tailings (see Fig. 2 and Table 1).

**Table 1:** Residue fraction (%) in the tailings

Fraction (%) in the residue	GMT	SMT
> 90%	Al, Sb, V	Al, Sb, V
81% to 90%	Cr, Li, Mo, Tl, Be	Cr, Li, Mo, Tl
70% to 79%	Fe, Mg, Zn	As, U
50% to 69%	As, Mn, Ni, U	Be, Fe
30% to 49%	Ca, Cd, Co	Co, Mg, Mn, Ni, Pb, Se, Zn
20% to 29%	Pb, Se	Cd
1% to 10%	Cu	Ca, Cu

## Discussion

The extraction conditions using the single-step precipitation extraction approach have mobilized Ca and Mg but barely dissolved any other metals from the tailings. The high release of Ca and Mg likely increases pH and the hardness of natural waters. Mo and Se were also dissolved in precipitation conditions, which could raise concerns for water quality. With more acidic extractants (BCR step 1), the two tailings released more metals at higher concentrations, particularly Ca, compared to precipitation extraction. Other metals dissolved in these conditions are Cd, Cu, and Zn. Under reducing condition (BCR step 2), high concentrations of Fe were observed together with Pb in both of the tailings, and higher amounts of Ni and Mn were found in SMT compared to GMT. Under oxidizing condition (BCR step 3), Fe was still detected at high concentration. Additionally, Co, Cu, Ni, and Se were also observed from both tailings. Moreover, higher ratio of As, Cd, and Zn were specific for SMT. The elements that remained over 80% in the residue were Al, Be, Cr, Li, Mo, Sb, Tl, and V.

The metals that were potentially released in high concentration in all three steps were Ca, Cd, Co, Cu, Fe, Mg, Mn, Ni, Pb, and Zn. However, some metals were dependent upon the type of tailings, such as in SMT that was released at high proportions in all three extraction steps. Concentrations of the toxic metals released by the BCR extraction are indicating concern to aquatic environment.

## Conclusion

- Mobility characteristics of the two tailings are as follows: precipitation and acid extraction dissolved large amounts of Ca and Mg. Furthermore, Ca and Mg controlled the dissolution of trace metals, including Cd, Cu, Mn, and Zn. Meanwhile, Fe as clay particle controlled the dissolution of Ni, and Pb, and Fe as mineral sulfide for Cu, Ni, Se, U, and Zn.
- Higher concentrations of toxic metals were observed in SMT, implying the source of release is tailing-dependent.
- The BCR sequential extraction can be used as an assessment tool to predict the release of toxic metals from solid mine wastes. The BCR sequential extraction was more aggressive extraction compared to a weak acid precipitation. The extraction to mine wastes could be improved by (1) pre-extraction with precipitation (targeting exchangeable and water-soluble minerals), (2) repeating BCR step 1 and step 3 extraction twice to ensure the complete dissolution of carbonate and sulfide minerals, respectively, and (3) analyzing rinse solution from each step to ensure no loss of metals and improve the extraction recovery.
- High concentrations of Ca and Fe in the tailings could carry over to the next extraction step and cause an incomplete extraction, resulting in a misinterpretation of mineral phase dissolution.

## Supplementary Information

Below is the link to the electronic supplementary material.

Supplementary file1 (DOCX 7.62 MB)
