# Supplementary material for: Investigation of metal mobility in gold and silver mine tailings by single-step and sequential extractions
Source: Environ Monit Assess. 2022 May 12;194(6):423. doi: 10.1007/s10661-022-10054-3 (PMC9098622; doi:10.1007/s10661-022-10054-3)
Supplement: Supplementary file 1 — Supplementary file1 (DOCX 7.62 MB) [file 10661_2022_10054_MOESM1_ESM.docx]

**Supplementary information**

**Investigation of metal mobility in gold and silver mine tailings by single-step and sequential extractions**

Paramee Kumkrong^1^*, Eben Dy^1^, Daniel D. Tyo^1^, Cindy Jiang^1^, Indu Gedara Pihilligawa^1^, David Kingston^1^, and Patrick H. J. Mercier^2^

^1^ National Research Council Canada, 1200 Montreal Road, Ottawa, Ontario Canada, K1A 0R6

^2^ Corem, Quebec, Canada

*Corresponding author: paramee.kumkrong@nrc-cnrc.gc.ca

ORCID : 0000-0002-9281-9974

**BCR extraction reagent**

Extraction reagent 1: 0.11 mol L^-1^ acetic acid

Pipetting 5.7 mL of 100 % v/v acetic acid (CAS 64-19-7) into DIW and make up to 1 L

Extraction reagent 2: 0.5 mol L^-1^ hydroxylamine hydrochloride

Weigh 34.75 g hydroxylamine hydrochloride (CAS 5470-11-1) in 400 mL of DIW, add 25 mL of 2 mol L^-1^ HNO_3_ and make up to 1 L with DIW (freshly prepared)

Extraction 3: 30 % w/w hydrogen peroxide (H_2_O_2_, CAS 7722-84-1 use as-it) and 1 mol L^-1^ ammonium acetate at pH 2.0

Weigh 77.08 g ammonium acetate (CAS 631-61-8) in 800 mL of DIW, add dropwise 69% w/w HNO_3_ to obtain pH 2.0 ± 0.1, and add DIW to 1 L

### **Total digestion procedure for tailings and residue after extraction**

Three acids including 69 % w/w nitric acid (HNO_3_), 37 % w/w hydrochloric acid (HCl) and 48 % w/w hydrofluoric acid (HF), were used for a microwave digestion (Anton Paar Multiwave PRO with eight digestion tubes, Anton Paar GmbH, Austria).

10 mL of HNO_3_, 5 mL of HCl and 5 mL of HF were added to a Teflon digestion vessel containing 0.25 g of sample. The mixture was left in the fume hood for 1 h for pre-digestion. The microwave program was set at 1,200 Watts with a 15 min ramp to 200°C and a 30 min hold at 200°C. After the vessels cooled to room temperature, excess amount of HF was destroyed by adding 10 mL of 6 % (w/v) boric acid (H_3_BO_3_) into each vessel. The vessels were placed in the microwave at 1,200 Watts with a 10-min ramp to 170°C and a 10-min hold at 170°C. Following the run, the samples were placed in a hot block at 96 ± 5°C and reduced to 5 mL volume. The concentrated samples were diluted to 50 g using 2 % (v/v) HNO_3_.

Figure S1: Percent recovery of elements and expanded uncertainty in comparison to certified value and associated uncertainty of PACS-3

Table S1: Total elemental results of PACS-3 in this study compared to certified value (mg kg^-1^)

| PACS-3 | Concentration of element (mg kg^-1^) | | | | |
| --- | --- | --- | --- | --- | --- |
|  | Certified | U (k=2) | Found | U (k=2) | Recovery (%) |
| Al | 65,800 | 1200 | 58,340 | 8,432 | 89 |
| As | 30.3 | 2.4 | 24.6 | 4.6 | 81 |
| Be | 1.06 | 0.12 | 1.03 | 0.18 | 97 |
| Ca | 18,900 | 1200 | 18,486 | 849 | 98 |
| Cd | 2.23 | 0.16 | 2.49 | 0.53 | 111 |
| Cr | 90.6 | 4 | 86.0 | 7.3 | 95 |
| Cu | 326 | 10 | 292 | 13 | 90 |
| Fe | 41,060 | 640 | 37,361 | 2,831 | 91 |
| Li | 31.9 | 4.2 | 36.9 | 3.7 | 116 |
| Mg | 14,020 | 580 | 14,485 | 2,079 | 103 |
| Mn | 432 | 16 | 409 | 32 | 95 |
| Ni | 39.5 | 2.2 | 41.7 | 4.6 | 106 |
| Pb | 188 | 7.4 | 179 | 19 | 95 |
| S | 11,700 | 4,400 | 11,897 | 197 | 102 |
| Sb | 14.7 | 2.2 | 11.2 | 1.6 | 76 |
| V | 129 | 8 | 127 | 4 | 98 |
| Zn | 376 | 12 | 352 | 22 | 94 |

Figure S2: Percent recovery (blue bar) and relative standard deviation (I-shaped line overlaid on blue bar) from the BCR extraction step 1, 2 and 3, and the residue digestion in comparison to the certified value and associated uncertainty (I-shaped line beside each blue bar) of BCR 701 CRM.

Table S2: Mass fraction (mg kg^-1^) of elements in GMT; total, single-step and BCR extraction and recovery

| GMT element | Total element | | Single-step extraction | | BCR step 1 | | BCR step 2 | | BCR step 3 | | Residue | | Σ(BCRs + residue) | Recovery |
| --- | --- | --- | --- | --- | --- | --- | --- | --- | --- | --- | --- | --- | --- | --- |
|  | mg/kg | SD | mg/kg | SD | mg/kg | SD | mg/kg | SD | mg/kg | SD | mg/kg | SD | mg/kg | (%) |
| Al | 52,321 | 901 | 0.801 | 0.262 | 143 | 2 | 856 | 2 | 713 | 32 | 51,910 | 3,433 | 53,623 | 102 |
| As | 1.23 | 0.05 | 0.002 | 0.001 | 0.032 | 0.008 | 0.316 | 0.011 | 0.168 | 0.033 | 0.887 | 0.074 | 1.40 | 114 |
| Be | 2.39 | 0.03 | ≤ 0.01 | nd | 0.047 | 0.001 | 0.085 | 0.009 | 0.117 | 0.038 | 1.43 | 0.15 | 1.68 | 70 |
| Ca | 19,976 | 1,047 | 2,311 | 79 | 10,216 | 364 | 1,758 | 22 | 77.1 | 5.4 | 8,980 | 451 | 21,032 | 105 |
| Cd | 0.205 | 0.062 | ≤ 0.01 | 0.000 | 0.065 | 0.003 | 0.022 | 0.003 | 0.021 | 0.004 | 0.064 | 0.011 | 0.172 | 84 |
| Co | 29.6 | 1.5 | 0.014 | 0.006 | 1.84 | 0.09 | 1.67 | 0.03 | 12.4 | 1.0 | 11.8 | 0.5 | 27.7 | 93 |
| Cr | 249 | 7 | ≤ 0.01 | nd | 8.30 | 0.52 | 14.5 | 0.2 | 8.12 | 0.12 | 183 | 5 | 214 | 86 |
| Cu | 103 | 8 | 0.102 | 0.020 | 57 | 2 | 16.4 | 0.6 | 26.7 | 2.7 | 2.16 | 0.63 | 103 | 99 |
| Fe | 37,266 | 282 | ≤ 0.02 | nd | 972 | 42 | 3,300 | 210 | 6,850 | 552 | 26,024 | 1,675 | 37,146 | 100 |
| Li | 23.8 | 3.2 | 0.061 | 0.006 | 0.280 | 0.062 | 0.71 | 0.04 | 1.89 | 0.03 | 22.8 | 3.2 | 25.7 | 120 |
| Mg | 8,085 | 3,520 | 73.3 | 3.3 | 713 | 16 | 549 | 8 | 537 | 29 | 6,749 | 302 | 8,549 | 106 |
| Mn | 571 | 27 | 1.64 | 0.203 | 225 | 4 | 29.4 | 2.0 | 23.8 | 4.0 | 356 | 12 | 635 | 111 |
| Mo | 12.6 | 0.9 | 0.148 | 0.002 | 0.005 | 0.001 | 0.123 | 0.006 | 0.817 | 0.156 | 9.6 | 0.8 | 10.6 | 84 |
| Ni | 78.1 | 8.4 | 0.017 | 0.004 | 2.60 | 0.05 | 6.85 | 0.09 | 14.4 | 1.5 | 42.9 | 2.0 | 66.8 | 85 |
| Pb | 32.3 | 6.2 | ≤ 0.01 | nd | 3.86 | 0.24 | 16.6 | 1.5 | 1.202 | 0.159 | 6.48 | 0.29 | 28.2 | 87 |
| Sb | 0.331 | 0.027 | 0.003 | 0.001 | 0.003 | 0.001 | 0.012 | 0.001 | 0.007 | 0.001 | 0.214 | 0.013 | 0.236 | 71 |
| Se | 0.426 | 0.052 | 0.007 | 0.001 | ≤ 0.02 | nd | 0.028 | 0.002 | 0.397 | 0.034 | 0.110 | 0.039 | 0.535 | 126 |
| Tl | 0.502 | 0.067 | ≤ 0.01 | nd | 0.003 | 0.001 | 0.030 | 0.001 | 0.035 | 0.003 | 0.436 | 0.029 | 0.503 | 100 |
| U | 2.34 | 0.35 | 0.005 | 0.002 | 0.316 | 0.007 | 0.154 | 0.006 | 0.423 | 0.020 | 1.16 | 0.06 | 2.05 | 88 |
| V | 105 | 4 | ≤ 0.02 | nd | 0.442 | 0.010 | 2.88 | 0.07 | 2.34 | 0.12 | 91.3 | 4.4 | 96.9 | 92 |
| Zn | 86.5 | 7.2 | 1.52 | 0.28 | 6.71 | 0.28 | 5.19 | 0.42 | 5.31 | 0.75 | 58.8 | 2.0 | 76.0 | 88 |
| C | 4,400 | 100 | nd | nd | nd | nd | nd | nd | nd | nd | nd | nd | nd | nd |
| H | 1,640 | 178 | nd | nd | nd | nd | nd | nd | nd | nd | nd | nd | nd | nd |
| N | 100 | 10 | nd | nd | nd | nd | nd | nd | nd | nd | nd | nd | nd | nd |
| S | 12,700 | 185 | nd | nd | nd | nd | nd | nd | nd | nd | 3,750 | 54 | nd | nd |

nd: not determined

Table S3: Concentration (mg kg^-1^) of elements from SMT before and after single-step and BCR extraction

| SMT  elements | Total element | | Single-step extraction | | BCR step 1 | | BCR step 2 | | BCR step 3 | | Residue | | Σ(BCRs + residue) | Recovery |
| --- | --- | --- | --- | --- | --- | --- | --- | --- | --- | --- | --- | --- | --- | --- |
|  | mg/kg | SD | mg/kg | SD | mg/kg | SD | mg/kg | SD | mg/kg | SD | mg/kg | SD | mg/kg | (%) |
| Al | 56,242 | 2,539 | 0.528 | 0.191 | 180 | 4 | 1,840 | 27 | 1,056 | 24 | 51,786 | 4,516 | 54,863 | 98 |
| As | 2,171 | 267 | 0.149 | 0.013 | 60 | 3 | 153 | 4 | 313 | 36 | 1,293 | 44 | 1,820 | 84 |
| Be | 1.84 | 0.30 | ≤ 0.01 | nd | 0.226 | 0.002 | 0.339 | 0.083 | 0.113 | 0.003 | 0.797 | 0.098 | 1.47 | 80 |
| Ca | 11,878 | 974 | 1,786 | 76 | 7,102 | 157 | 3,016 | 43 | 82.8 | 1.9 | 387 | 38 | 10,588 | 89 |
| Cd | 4.77 | 0.09 | 0.006 | 0.001 | 1.39 | 0.02 | 0.342 | 0.017 | 1.60 | 0.13 | 1.023 | 0.081 | 4.35 | 91 |
| Co | 33.7 | 4.8 | 0.015 | 0.004 | 3.45 | 0.03 | 2.57 | 0.04 | 8.65 | 0.62 | 12.7 | 0.2 | 27.4 | 81 |
| Cr | 57.7 | 1.9 | ≤ 0.01 | nd | 1.00 | 0.03 | 4.31 | 0.05 | 1.63 | 0.04 | 38.1 | 0.7 | 45.1 | 78 |
| Cu | 1,041 | 37 | 0.145 | 0.033 | 223 | 7 | 147 | 2 | 541 | 29 | 82.1 | 4.9 | 992 | 95 |
| Fe | 60,186 | 1,206 | ≤ 0.02 | nd | 1,594 | 16 | 9,501 | 618 | 16,637 | 935 | 27,822 | 298 | 55,555 | 92 |
| Li | 36.6 | 4.2 | 0.101 | 0.008 | 0.800 | 0.033 | 1.73 | 0.11 | 1.29 | 0.22 | 23.2 | 3.3 | 27 | 74 |
| Mg | 2,826 | 518 | 109 | 5 | 819 | 20 | 416 | 8 | 316 | 11 | 940 | 20 | 2,490 | 88 |
| Mn | 758 | 43 | 2.92 | 0.21 | 258 | 5 | 146 | 5 | 57.3 | 5.9 | 254 | 17 | 716 | 94 |
| Mo | 12.2 | 1.7 | 1.83 | 0.10 | 0.018 | 0.002 | 0.923 | 0.074 | 0.646 | 0.100 | 8.07 | 0.59 | 9.65 | 79 |
| Ni | 48.8 | 2.3 | 0.015 | 0.004 | 6.75 | 0.40 | 11.1 | 0.2 | 9.6 | 1.3 | 15.6 | 1.2 | 43.1 | 88 |
| Pb | 358 | 30 | ≤ 0.01 | nd | 28 | 0.665 | 172 | 6 | 6.31 | 0.16 | 138 | 22 | 344 | 96 |
| Sb | 285 | 21 | 2.79 | 0.15 | 2.04 | 0.04 | 10.1 | 0.7 | 3.30 | 0.18 | 265 | 21 | 281 | 99 |
| Se | 0.969 | 0.067 | 0.033 | 0.002 | 0.030 | 0.008 | 0.093 | 0.010 | 0.350 | 0.051 | 0.326 | 0.052 | 0.779 | 84 |
| Tl | 1.84 | 0.21 | ≤ 0.01 | nd | 0.004 | 0.001 | 0.036 | 0.001 | 0.018 | 0.003 | 1.77 | 0.13 | 1.82 | 99 |
| U | 2.87 | 0.62 | 0.004 | 0.001 | 0.237 | 0.004 | 0.154 | 0.004 | 0.153 | 0.004 | 1.82 | 0.17 | 2.36 | 82 |
| V | 154 | 5 | <0.02 | nd | 0.264 | 0.006 | 3.61 | 0.14 | 1.13 | 0.05 | 129 | 21 | 134 | 87 |
| Zn | 646 | 12 | 0.836 | 0.170 | 111 | 2 | 63.5 | 6.1 | 214 | 6 | 223 | 5 | 612 | 95 |
| C | 3,500 | 57 | nd | nd | nd | nd | nd | nd | nd | nd | nd | nd | nd | nd |
| H | 3,550 | 386 | nd | nd | nd | nd | nd | nd | nd | nd | nd | nd | nd | nd |
| N | 300 | 12 | nd | nd | nd | nd | nd | nd | nd | nd | nd | nd | nd | nd |
| S | 29,440 | 422 | nd | nd | nd | nd | nd | nd | nd | nd | 12,790 | 183 | nd | nd |

nd: not determined

Figure S3: BSE image of GMT tailings a-1) 200 µm and a-2) 50 µm; GMT after single-step extraction b-1) 200 µm and b-2) 50 µm; and GMT after BCR extraction c-1) 200 µm and c-2) 20 µm


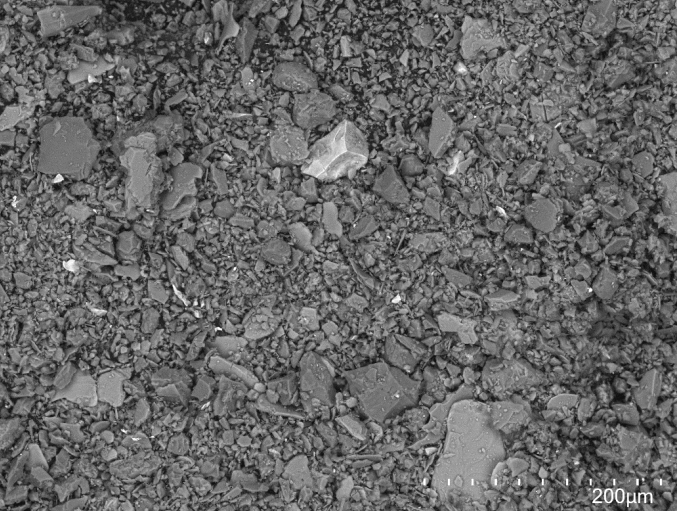

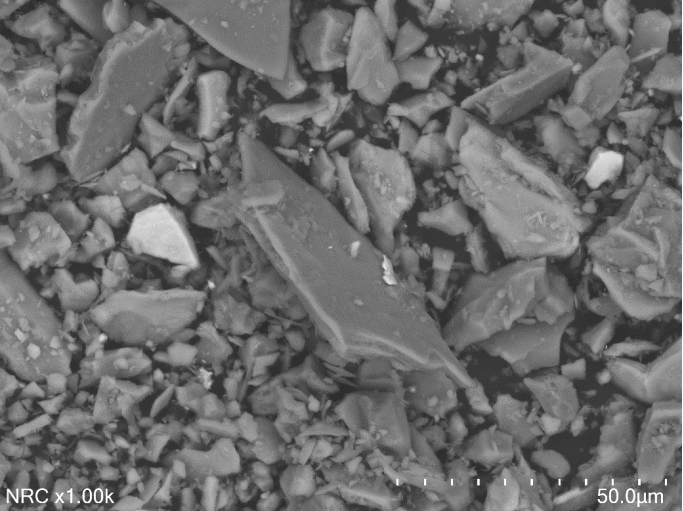

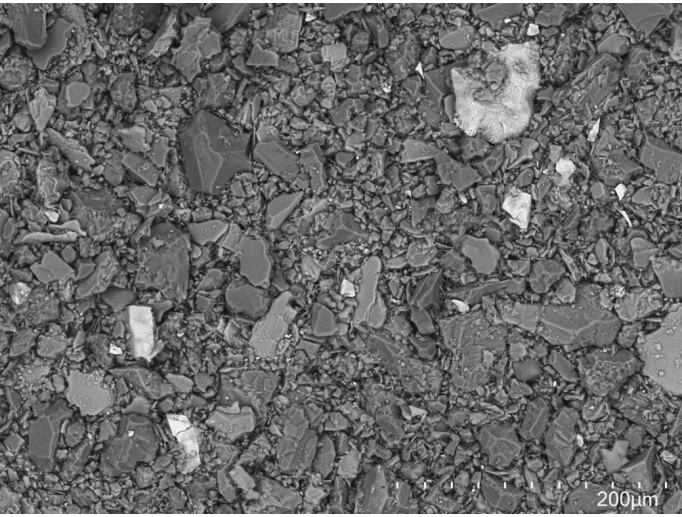

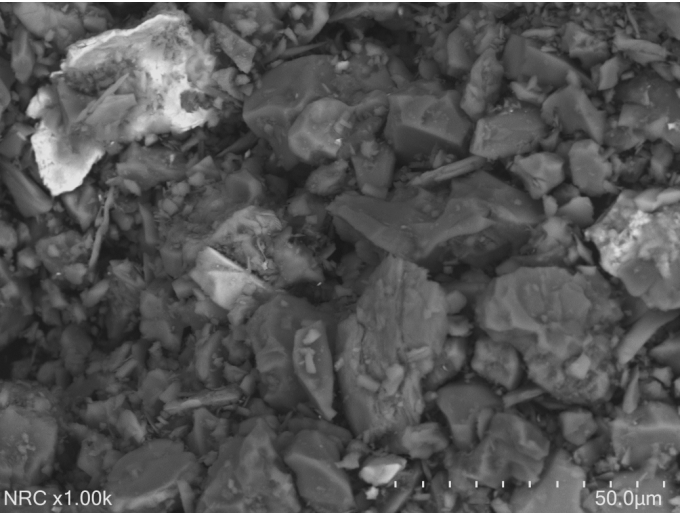

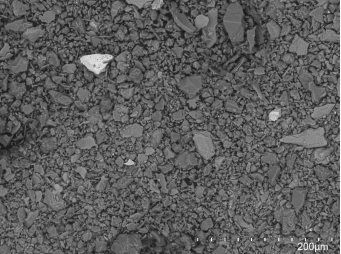

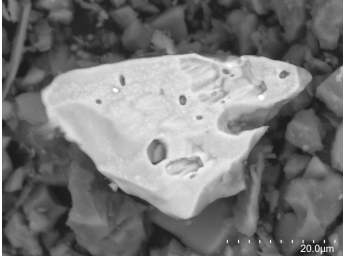


**a-1)**

**a-2)**

**b-1**

**b-2)**

**c-1)**

**c-2)**

Figure S4: BSE image of SMT sample at SMT tailings a-1) 200 µm and a-2) 50 µm; SMT after single extraction b-1) 200 µm and b-2) 50 µm; and SMT after BCR extraction c-1) 200 µm and c-2) 50 µm


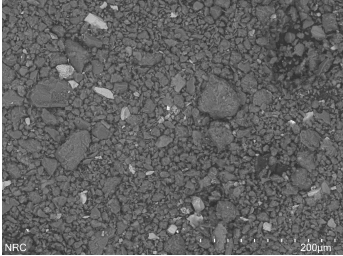

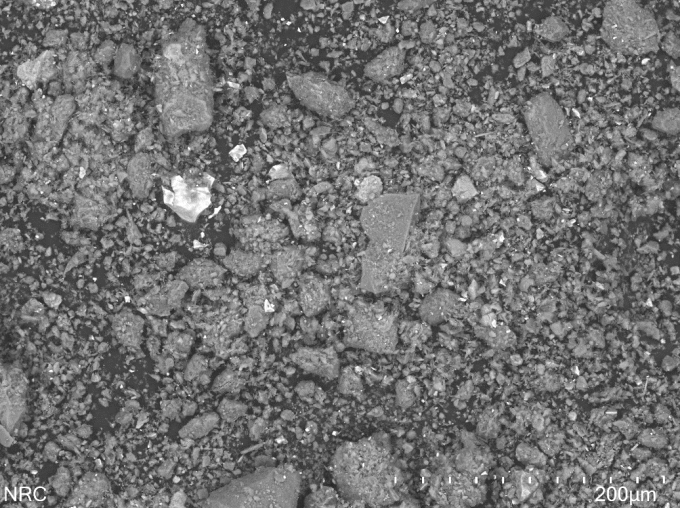

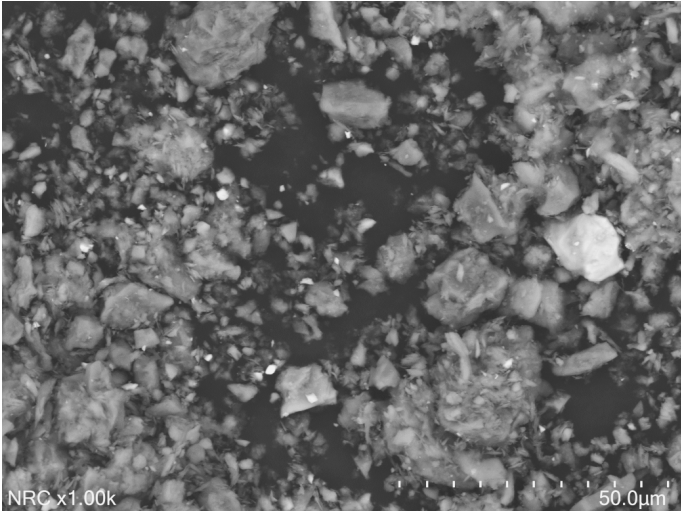

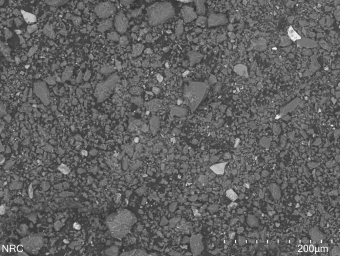

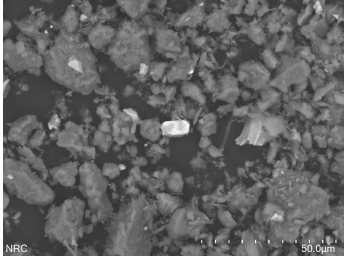

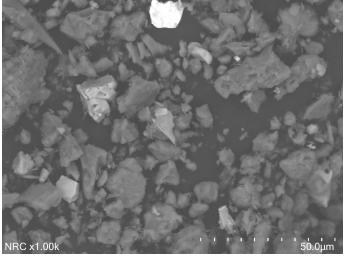


**a-1)**

**a-2)**

**b-1)**

**b-2)**

**c-1)**

**c-2)**

b)

a)

a)

Table S4: Percent fraction of elements from GMT and SMT by single and BCR extractions

| Element | Single extraction (%) GMT | BCR fraction (%) in GMT | | | | Single extraction (%) SMT | BCR fraction (%) in SMT | | | |
| --- | --- | --- | --- | --- | --- | --- | --- | --- | --- | --- |
|  |  | Step 1 | Step 2 | Step 3 | Residue |  | Step 1 | Step 2 | Step 3 | Residue |
| Al | 0.0 | 0.3 | 2 | 1 | 97 | 0.0 | 0.3 | 3 | 2 | 94 |
| As | 0.2 | 2 | 23 | 12 | 63 | 0.0 | 3 | 8 | 17 | 71 |
| Be | 0.1 | 3 | 5 | 7 | 85 | 0.1 | 15 | 23 | 8 | 54 |
| Ca | 12 | 49 | 8 | 0.4 | 43 | 15 | 67 | 28 | 0.8 | 4 |
| Cd | 0.1 | 38 | 13 | 12 | 37 | 0.1 | 32 | 8 | 37 | 23 |
| Co | 0.0 | 7 | 6 | 45 | 42 | 0.0 | 13 | 9 | 32 | 46 |
| Cr | 0.0 | 4 | 7 | 4 | 86 | 0.0 | 2 | 10 | 4 | 85 |
| Cu | 0.1 | 56 | 16 | 26 | 2 | 0.0 | 22 | 15 | 54 | 8 |
| Fe | 0.0 | 3 | 9 | 18 | 70 | 0.0 | 3 | 17 | 30 | 50 |
| Li | 0.3 | 1 | 3 | 7 | 89 | 0.3 | 3 | 6 | 5 | 86 |
| Mg | 0.9 | 8 | 6 | 6 | 79 | 4 | 33 | 17 | 13 | 38 |
| Mn | 0.3 | 35 | 5 | 4 | 56 | 0.4 | 36 | 20 | 8 | 36 |
| Mo | 1.2 | 0.1 | 1 | 8 | 91 | 15 | 0.2 | 10 | 7 | 84 |
| Ni | 0.0 | 4 | 10 | 22 | 64 | 0.0 | 16 | 26 | 22 | 36 |
| Pb | 0.0 | 14 | 59 | 4 | 23 | 0.0 | 8 | 50 | 2 | 40 |
| Sb | 1.0 | 0.7 | 5 | 3 | 90 | 1.0 | 0.7 | 4 | 1 | 94 |
| Se | 2 | nd | 5 | 74 | 21 | 3 | nd | 12 | 45 | 42 |
| Tl | 0.1 | 0.6 | 6 | 7 | 87 | 0.0 | 0.2 | 2 | 10.8 | 97 |
| U | 0.2 | 15 | 8 | 21 | 56 | 0.1 | 10 | 7 | 6 | 77 |
| V | nd | 0.5 | 3 | 2 | 94 | nd | 0.2 | 3 | 0.8 | 96 |
| Zn | 1.8 | 9 | 7 | 7 | 77 | 0.1 | 18 | 10 | 35 | 36 |

nd: not determined
